# A reporter mouse for non-invasive detection of toll-like receptor ligands induced acute phase responses

**DOI:** 10.1038/s41598-019-55281-w

**Published:** 2019-12-13

**Authors:** Chun-Fang Huang, Shang-Yi Chiu, Hung-Wen Huang, Bing-Ho Cheng, Hsiu-Min Pan, Wei-Lun Huang, Hsiao-Hui Chang, Chia-Chi Liao, Si-Tse Jiang, Yu-Chia Su

**Affiliations:** National Laboratory Animal Center, National Applied Research Laboratories, Taipei, Taiwan

**Keywords:** Genetic engineering, Bioluminescence imaging

## Abstract

The acute phase response (APR) is a systemic first-line defense against challenges including infection, trauma, stress, and neoplasia. Alteration of acute phase protein (APP) levels in plasma is the most important change during acute phase response. C-reactive protein (CRP), which increases dramatically during inflammation onset, is an indicator of inflammation. To monitor the process of APR, we generated human CRP promoter-driven luciferase transgenic (hCRP-Luc) mice to quantify the hCRP promoter activation *in vivo*. The naïve female hCRP-Luc mice express low basal levels of liver bioluminescence, but the naïve male hCRP-Luc mice do not. Thus, female hCRP-Luc mice are suitable for monitoring the process of APR. The liver bioluminescence of female hCRP-Luc mice can be induced by several toll-like receptor (TLR) ligands. The expression of liver bioluminescence was highly sensitive to endotoxin stimulation in a dose-dependent manner. On-off-on bioluminescence response was noted in female hCRP-Luc mice upon two endotoxin stimulations one month apart. The LPS-induced bioluminescence of the female hCRP-Luc mice was IL-6-mediated and associated with APP alpha-1-acid glycoprotein expression. In conclusion, the female hCRP-Luc mouse is a non-invasive, sensitive and reusable reporter tool for APR.

## Introduction

Acute phase response (APR) is a core function of the innate immune system to combat numerous immediate challenges, including infection, trauma, burns, neoplasia, or even strenuous exercise, heatstroke, labor, psychological stress, and psychiatric illness^1,2^. One of the most important changes during the APR is the alteration of acute phase protein (APP) levels in plasma^2^. Acute phase proteins are classified as positive APP (those induced by more than 25%) and negative APP (those repressed by more than 25%) in plasma during APR^2,3^. Positive APPs participate in and regulate several innate immunological responses, including the complement system, coagulation system, cytokine antagonists, and opsonization^2,4^. In clinical application, positive APPs are often used as indicators of inflammation. One of the most widely applied APPs in medical and biological research is C-reactive protein (CRP). The major role of CRP is to opsonize pathogens and dead cells to facilitate phagocytosis^5^, and it triggers the classical complement cascade^6,7^.

In humans, CRP expression increases rapidly within 6–8 hours during infection or inflammatory response and returns to the basal level quickly after inflammation is diminished^2,8^. CRP concentration in peripheral blood may increase 10,000 fold upon acute phase stimulus in humans^9^, making it an excellent biomarker for such conditions^10,11^. CRP is predominately synthesized by hepatocytes in response to pro-inflammatory cytokines, complements, and hormones^12–15^. Pro-inflammatory cytokines secreted by macrophages and monocytes, including IL-6, IL-1β, and TNFα, mediate CRP activation^16,17^. Among these cytokines, IL-6 is the major regulator of APP synthesis in adult human hepatocytes^14,16–19^. Both siltuximab (a chimeric anti-IL-6 antibody) and tocilizumab (a humanized anti-IL-6R antibody) can downregulate CRP production^20,21^. Other cytokines have been shown to have inconsistent effects in different reports. IL-1β was found to induce CRP production in several human hepatoma cell lines^19,22,23^. However, neither IL-1β- nor TNFα-stimulation induced CRP production in cultured human primary hepatocytes^14^. Moshage and his colleagues demonstrated that IL-1β-induced CRP production in human hepatocytes was mediated by IL-6^24^. Collectively, these findings indicate that the inflammatory response leading to CRP expression is an IL-6 dependent process.

CRP expression can also be affected by other non-inflammatory factors. Orchiectomy decreases circulating CRP concentrations without changing serum IL-1β and IL-6 concentrations in humans^25^, indicating that testicular hormones might also regulate CRP production. Indeed, testosterone could induce human CRP gene expression in hCRP transgenic mice^15^.

Unlike that in humans, CRP in mice is only a modest APP. The mouse serum CRP level is 5–9 mg/L at baseline and peaks at 17 mg/L after LPS injection^26^, indicating that the regulatory mechanisms in response to inflammatory stimulus of the two species are different. This species-specific pattern of CRP gene expression hinders the usage of CRP as a marker of inflammation in mice. Human CRP-expressing mice with the regulatory elements of the human gene have been generated using a cosmid-based strategy. hCRP expression in these mice is highly inducible and tissue specific under inflammatory stimulus^12,27^. These animals have been used for studying the mechanisms of APR and the biological functions of hCRP protein.

In this study, we generated a human CRP transgenic reporter mouse, using a bacterial artificial chromosome (BAC)-based approach to include regions of the human CRP promoter linked with luciferase cDNA sequences. *In vivo* imaging showed that this transgenic mouse exhibited a rapid, strong, and on-off-on bioluminescence signal upon several pattern recognition receptor (PRR) ligand stimulations, presumably *via* an IL-6 dependent pathway. This transgenic mouse has the advantage of reporting CRP gene instead of protein activation *in vivo* and may be used as a tool to monitor APR.

## Results

### Male sex hormone modulated hCRP expression in hCRP-Luc mice

**Figure 1:**
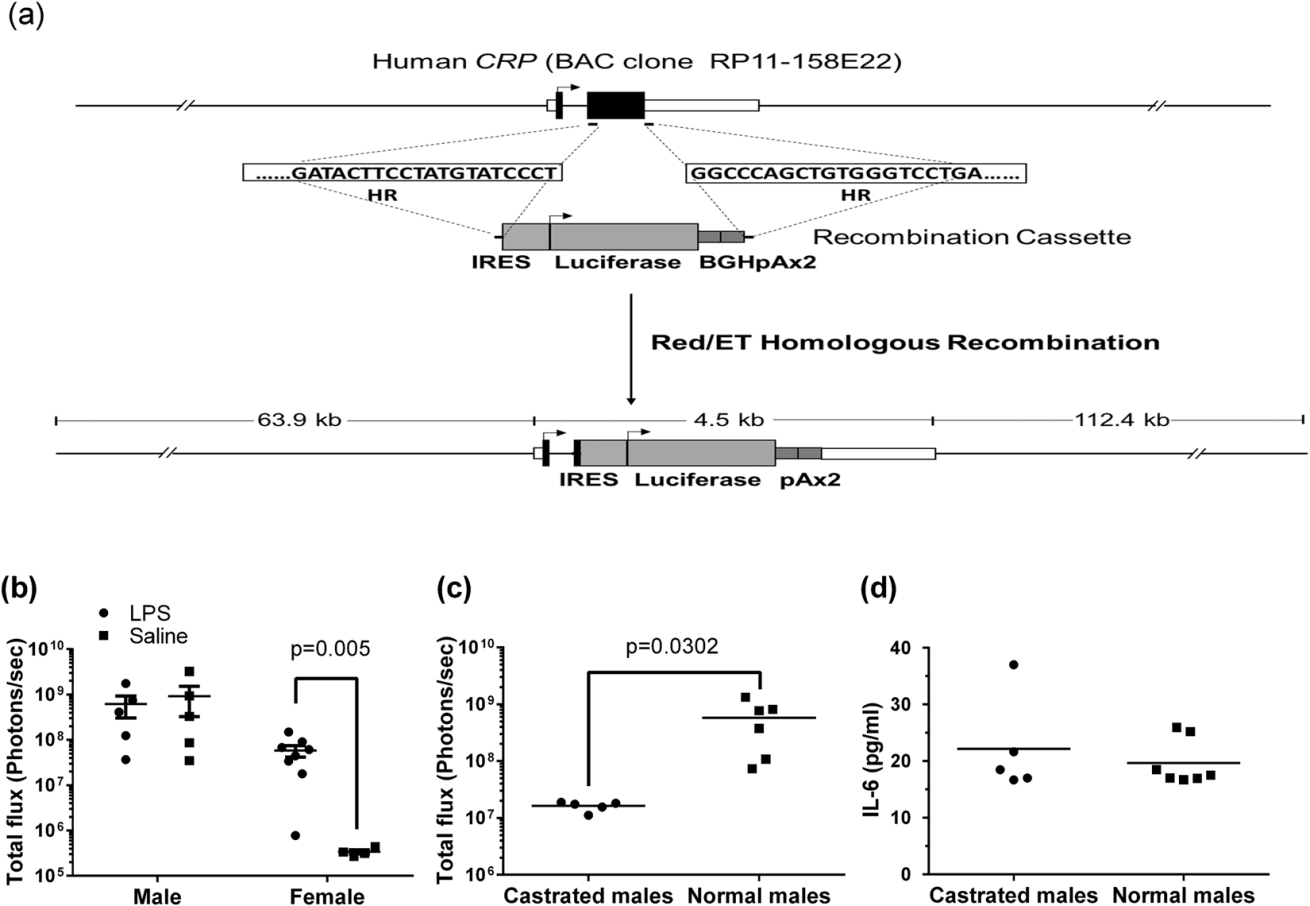
Male sex hormone modulated hCRP expression in hCRP-Luc mice. (**a**) Construction of human CRP-luciferase reporter. Recombination cassette containing the IRES-Luciferase-2x poly A and a 50-bp targeting homologous sequence was introduced into the BAC clone RP11-158E22 by Red/ET-mediated homologous recombination in XL-1 blue electroporation competent cells. The targeting homologous site was designed to replace the sequence between the exon 2 leader sequence and those sequences just behind the stop codon of human CRP with IRES-Luciferase-2x polyA. The end product was microinjected into mouse embryos at pronuclear stages. (**b**) hCRP-Luc mice were intraperitoneally injected with sterile PBS or LPS (100 ng/mouse). Mice were subjected to liver bioluminescence analysis by IVIS at 22 h post LPS injection. Data are means ± SEM (LPS-injected, male group, n = 5; PBS-injected, male group, n = 5; LPS-injected, female group, n = 8; PBS-injected, female group, n = 4). (**c**) Male hCRP-Luc mice (n = 5) were castrated and rested for 2 weeks, and the bioluminescence was compared with age-matched normal male hCRP-Luc mice by AmiHT (n = 7). Data are means ± SEM of two replicated experiments. (**d**) Serum IL-6 levels of the castrated (n = 5) and normal mice (n = 7) were determined by immunoassay.

The luciferase cDNA was introduced into the BAC construct containing the human CRP promoter as described in *Methods* (Fig. 1a). During our initial characterization of the hCRP-Luc mice, we observed that male mice exhibited a bioluminescence basal level higher than that of their female counterparts. We thus further characterized the differences in LPS-induced bioluminescence in male and female hCRP-Luc mice. Female hCRP-Luc mice showed a relatively low basal level (3.39 × 10^5^ photons/sec) with increased bioluminescence signals upon LPS stimulation (100 ng/mouse; 5.81 × 10^7^ photons/sec; Fig. 1b, *p = *0.005). The male mice had a high intrinsic luciferase activity (9.81 × 10^8^ photons/sec), which was already higher than that of LPS-stimulated female mice, and did not increase upon LPS stimulation (6.13 × 10^8^ photons/sec). Previous reports indicated that testosterone can modulate hCRP expression independent of IL-6^13,15,25^. We therefore evaluated the effect of castration on the bioluminescence intensity of male hCRP-Luc mice. As shown in Fig. 1c, normal male hCRP-Luc mice showed high bioluminescence signals (5.78 × 10^8^ photons/sec), whereas castrated male hCRP-Luc mice had a 98% reduction in bioluminescence (1.63 × 10^7^ photons/sec; *p* = 0.0302). Serum IL-6 levels between the castrated and normal male mice were not significantly different (Fig. 1d). The high basal level of bioluminescence signals in male hCRP-Luc mice was thus likely regulated by an IL-6-independent mechanism. This result confirmed our speculation, and female hCRP-Luc mice were chosen for the following experiments.

### Multiple TLR ligands induced liver bioluminescence in female hCRP-Luc mice with dose-dependent responses

**Figure 2:**
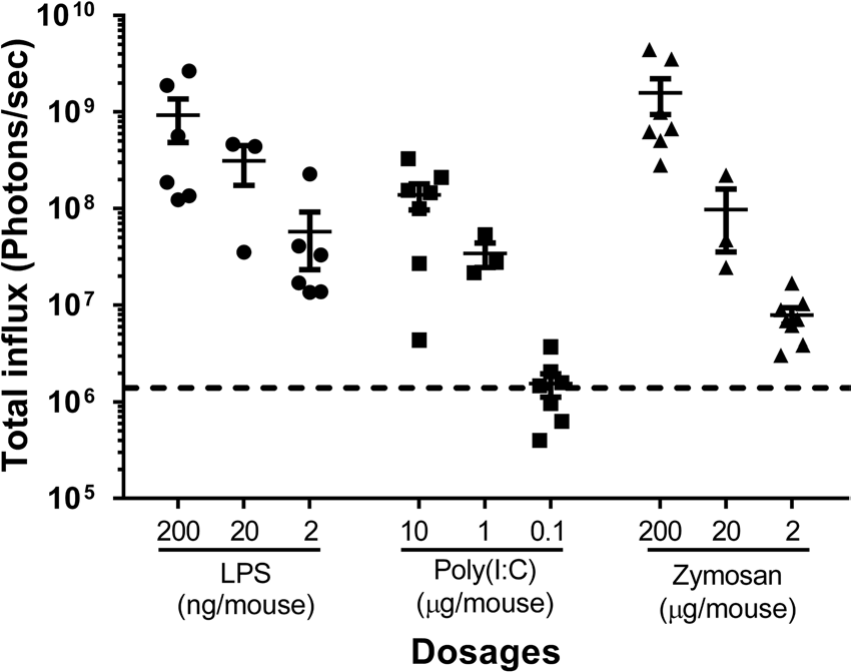
Titration of TLR-induced bioluminescence in female hCRP-Luc mice. Female hCRP-Luc mice were intraperitoneally injected with increasing doses of LPS (2, 20, 200 ng/mouse), poly(I:C) (0.1, 1, 10 μg/mouse), or zymosan (2, 20, 200 μg/mouse) and the bioluminescence of these mice was analyzed by AmiHT at 22 h post injection. Data are means ± SEM (n = 3–5) of two repeated experiments.

We next tested the sensitivity of bioluminescence induction of female hCRP-Luc mice in response to different TLR ligands (Fig. 2). We found that 2 ng/mouse of LPS induction resulted in an approximately 32.6-fold increase in bioluminescence intensity (5.75 × 10^7^ photons/sec) as compared to the basal level (1.76 × 10^6^ photons/sec). Ten-fold and 100-fold increases of the initial dosages resulted in additional 5.4-fold (3.11 × 10^8^ photons/sec) and 16.1 fold (9.23 × 10^8^ photons/sec) induction of the signal increase of the bioluminescence signal, respectively. Zymosan at the predetermined dosage of 2 μg/mouse resulted in a 4.6-fold increase of bioluminescence signal as compared to the basal level (8.11 × 10^6^ photons/sec). Increasing the dosages 10- and 100-fold resulted in further 55-fold (9.72 × 10^7^ photons/sec) and 897-fold (1.58 × 10^9^ photons/sec) increases in bioluminescence signal, respectively. Unlike LPS or zymosan, poly(I:C) at the predetermined dosage of 0.1 μg/mouse did not increase the bioluminescence signal (1.54 × 10^6^ photons/sec), and a 10-fold increase of the dosage resulted in a 19.4-fold increase in bioluminescence signal (3.42 × 10^7^ photons/sec). In addition, a 100-fold increase of the initial dosage resulted in a 78.4-fold increase in bioluminescence signal (1.38 × 10^8^ photons/sec). These results not only confirmed the responses of the female hCRP-Luc mice to different TLR ligands but also showed that these mice could respond to the ligands in a dose-dependent manner.

### LPS-induced bioluminescence and AGP acute phase protein expression was IL-6-dependent

**Figure 3:**
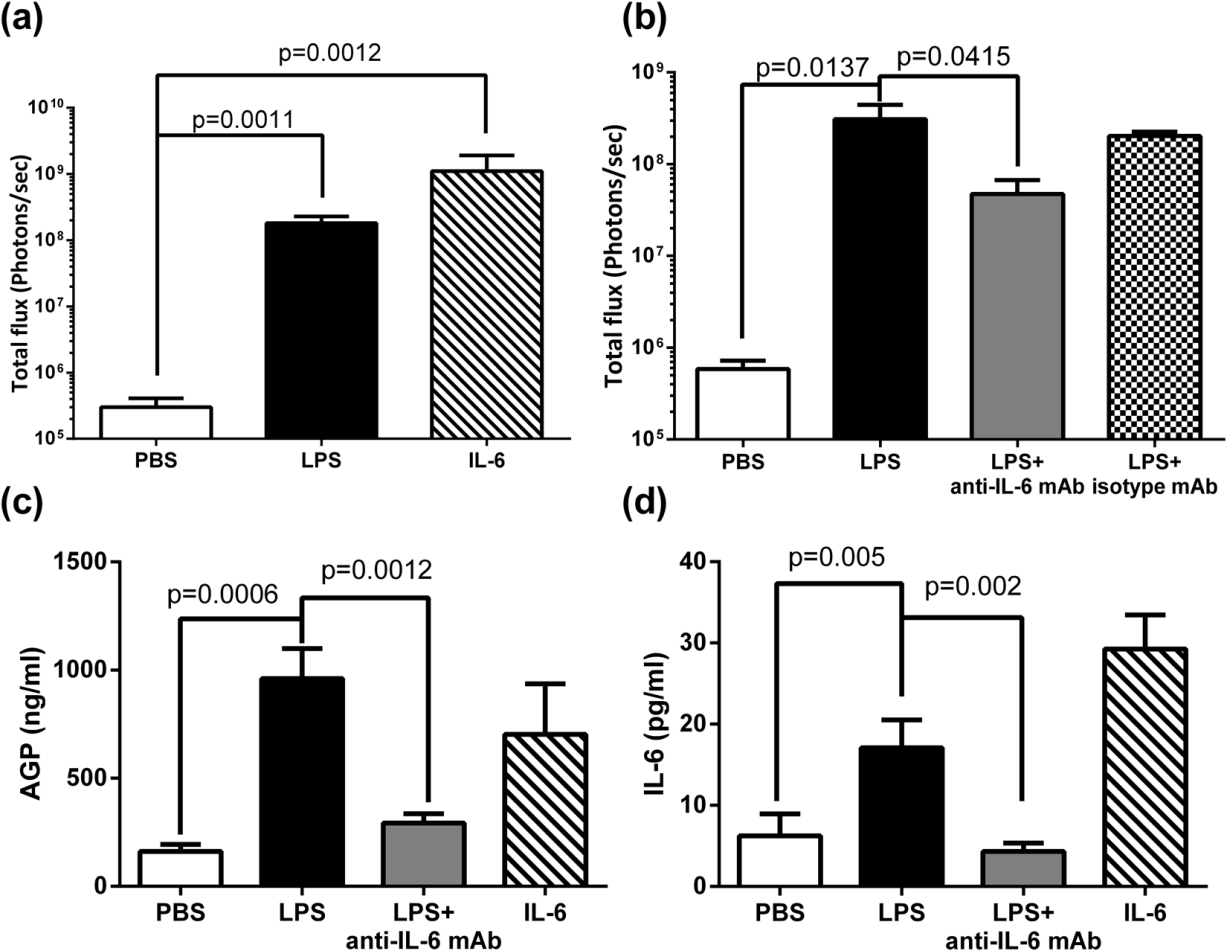
LPS-induced bioluminescence and AGP acute phase protein expression in female hCRP-Luc mice was mediated by IL-6. (**a**) Female hCRP-Luc mice were intraperitoneally injected with sterile PBS, LPS (100 ng/mouse), or IL-6 (1 μg/mouse). The liver bioluminescence was determined by IVIS at 22 h post LPS injection. Data are means ± SEM (n = 4–5) of two repeated experiments. (**b**) Female hCRP-Luc mice were intraperitoneally injected with sterile PBS, LPS (100 ng/mouse), co-injected with LPS (100 ng/mouse) and anti-IL-6 neutralized mAb (200 μg/mouse), or co-injected with LPS (100 ng/mouse) and isotype-matched irrelevant mAb (200 μg/mouse). The liver bioluminescence was determined by AmiHT at 22 h post LPS injection. Data are means ± SEM (n = 5–7) of two repeated experiments. (**c**) Serum samples were collected from mice described in (**a**) for determination of alpha-1-acid glycoprotein (AGP) and IL-6. Data are means ± SD (n = 4–5).

IL-6 is one of the most important cytokines mediating CRP induction^18,19^. A previous study using a human CRP transgenic model also showed that IL-6 is in part responsible for hCRP induction in mice^27^. To determine the involvement of IL-6 in our female hCRP-Luc mice, we blocked IL-6 signaling on the bioluminescence signal. We first evaluated whether IL-6 injection alone could trigger bioluminescence signal. IL-6 injection, similar to LPS, induced strong bioluminescence in the female hCRP-Luc mice (basal level: 3.02 × 10^5^ photons/sec; LPS: 1.81 × 10^8^ photons/sec, *p* = 0.001), and IL-6 (1.10 × 10^9^ photons/sec, *p* = 0.0012; Fig. 3a). Co-injection of anti-IL-6 antibody with LPS also resulted in an increase of bioluminescence signal from the basal level (4.72 × 10^7^ photons/sec and 5.83 × 10^5^ photons/sec, respectively; Fig. 3b). However, the magnitude was significantly lower as compared to mice injected with LPS only (3.10 × 10^8^ photons/sec, *p* = 0.0415) or with LPS and isotype matched antibody (2.03 × 10^8^ photons/sec). This indicated that LPS-induced bioluminescence was partially mediated by IL-6 and that other IL-6-independent pathways may exist in the female hCRP-Luc mice. We further analyzed whether AGP, a known IL-6-regulated APP, was also induced in this model^28^. Serum AGP levels were upregulated in female hCRP-Luc mice by LPS (961 pg/mL, *p* = 0.0006) and IL-6 (703 pg/mL) (Fig. 3c). Anti-IL-6 mAb treatment significantly repressed the LPS-induced serum AGP levels (294 pg/mL, *p* = 0.0012). Because IL-6 plays a central role in LPS-induced CRP and AGP, we also determined serum IL-6 levels (Fig. 3d). As expected, LPS induced serum IL-6 production (17.1 pg/mL, *p* = 0.005) and anti-IL-6 mAb treatment reduced the LPS-induced IL-6 levels to the basal level (4.32 pg/mL, *p* = 0.002). Significant changes was not detected for other APPs (e.g. adipsin, A2M, SAP) and TNF in sera, suggesting that they were not involved in the LPS induced acute phase response in the female hCRP-Luc mice (Figure S1).

### Human CRP-Luc mouse was a highly sensitive and reusable APR reporter

**Figure 4:**
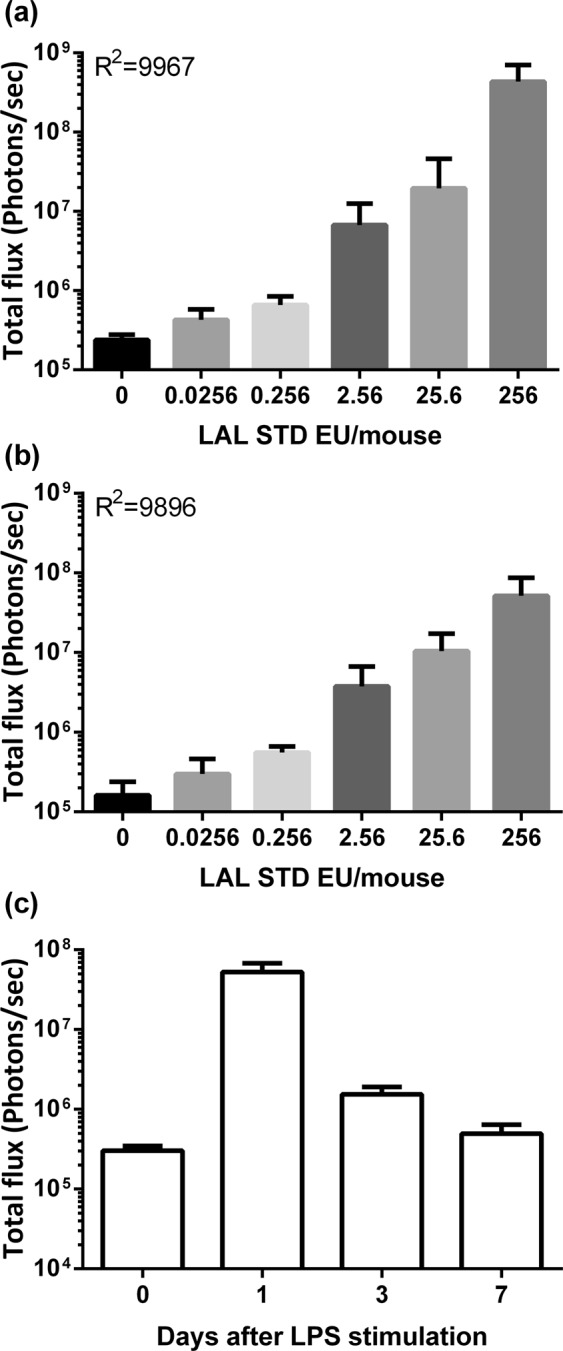
High sensitivity and re-inducible APR reporter of female hCRP-Luc mice. Serial dilutions of LAL standard samples in endotoxin-free saline were intraperitoneally injected into female hCRP-Luc mice (200 μL/mouse) twice one month apart. The liver bioluminescence was determined by IVIS at 22 h after each injection. (**a**) Bioluminescence activity after the first injection. (**b**) Bioluminescence activity after the second injection. Data are means ± SD (n = 3–5). (**c**) Female hCRP-Luc mice were intraperitoneally injected with LPS (100 ng/mouse). The liver bioluminescence was determined before (day 0) or at day 1, 3 and 7 post injection. Data are means ± SD (n = 5).

To evaluate the sensitivity of the female hCRP-Luc mouse, we compared its detection limit to limulus amebocyte lysate (LAL) assay, the current standard pyrogen test. LAL diluted standards (0.0256–256 EU) were injected intraperitoneally to female hCRP-Luc mice, and *in vivo* luciferase assays were performed at 22 h after injection. The results showed a dose-dependent induction with a good correlation (R^2^ = 0.9967; Fig. 4a). Notably, the bioluminescence of the lowest dose of the LAL standards (0.0256 EU/mouse) was still significantly higher than that of the control, indicating the female hCRP-Luc mice exerted a potential low detection limit. The same animals of this study were re-stimulated with LAL standards one month after the first treatment (Fig. 4b). The repeated stimulation also showed good correlation of dose-dependent luciferase activity (R^2^ = 0.9896). To determine the clearance time of luciferase, female hCRP-Luc mice were stimulated with LPS, and luciferase responses were determined 1, 3 and 7 days after stimulation (Fig. 4c). In female hCRP-Luc mice, strong bioluminescence (5.27 × 10^7^ photons/sec) was induced at day 1 post stimulation and decreased to near the baseline level at day 7 (4.97 × 10^5^ photons/sec). This indicated that the bioluminescence of female hCRP-Luc mice can return to the baseline after stimulant removal and suggested that repeated stimulation is possible.

## Discussion

In this study, we generated human CRP promoter-driven luciferase transgenic mice. Only female hCRP-Luc mice showed increased expression of liver bioluminescence signals with dose-dependent responses after TLR-ligand stimulations. Male hCRP-Luc mice showed high basal levels of bioluminescence signals, which could be reduced by castration. We also proved that the LPS-induced bioluminescence was IL-6-mediated and parallel to acute phase protein AGP expression. In addition, the female hCRP-Luc mice showed high sensitivity to LAL standard endotoxin stimulation (0.0256 EU/mouse) and good dose-dependent correlation with LAL endotoxin. The endotoxin-induced bioluminescence returned to the baseline value 7 days post-stimulation and could be induced repeatedly.

In our hCRP-Luc mice, the CRP coding sequence was replaced with a luciferase reporter gene, and APR was quantitated based on luminescence instead of CRP protein production. Two methods have been commonly used for APR evaluation: blood APP quantification and body temperature measurement. APP quantification requires blood sampling which may increase risks of minor APR induction at the puncture site^29^, and detection of CRP or SAP by immunoassays would increase variability in the evaluation of APR. Changes in body temperature, as being used in the rabbit pyrogen test, can be used as sign of early stage inflammation. Pharmaceutical products containing 13.81 EU/mL endotoxin can induce a body temperature rise of 0.5 °C in rabbit pyrogen tests^30^. However, the long-term restraining of animals not only raises animal welfare issues but also affects the results of the pyrogen test^31^. Moreover, it is hard to apply the same method to laboratory mice. Measuring APR with our female hCRP-luc mice not only eliminates all the aforementioned difficulties, the fact that the mice could be repeatedly induced by TLR ligands also make them suitable for detection of non-continuous APR stimulations.

Our female hCRP-Luc mice would be a good model for studying IL-6- or non-IL-6-regulated CRP induction. Several studies have indicated that IL-6 is the principle inducer of CRP gene expression in humans; IL-1β, TNF-α and complements synergize with IL-6^18,23,32–34^. Previous reports have shown that IL-6 production in mice after LPS-stimulation is extremely poor compared to that in humans^35^, and IL-1 is a stronger pyrogen than IL-6 in mice^36^. However, one study demonstrated that IL-1β-induced CRP production in human hepatocytes was mediated by IL-6^24^, so we focus on IL-6-stimulated CRP production in our study. We found that LPS could induce a low level of IL-6 production (<30 pg/mL in sera, Fig. 3d), and at this level, bioluminescence expression in female hCRP-Luc mice was observed. We attributed this hCRP-Luc expression to IL-6, for we found that blockage of IL-6 using anti-IL-6 antibody significantly reduced the LPS-induced bioluminescence of hCRP-Luc mice. On the other hand, blocking IL-6 did not fully eliminate either LPS-induced bioluminescence or AGP expression (Fig. 3b,c), indicating the possible involvement of non-IL-6-mediated regulation. These results are in agreement with previous findings of PRR-induced, multiple pathway-mediated APPs^16,17,19^.

CRP is a marker of inflammation in both male and female patients. Thus, a major limitation of our hCRP-Luc mice is that only female mice could be used for detection of APR. Median levels and 95 percentiles of plasma testosterone levels are 0.19–12.18 ng/mL in mature male mice, which is similar to the normal range in human males^37^. Our male hCRP-Luc mice showed high levels of autonomous bioluminescence signal, which could be reduced by 98% *vi*a castration. This finding is in line with a previous report that orchiectomy decreased circulating CRP concentrations without changing serum IL-1β and IL-6 concentrations in humans^25^. Therefore, it might indicate that testicular hormones are involved in the regulation of hCRP promoter driven bioluminescence. In addition, it has been reported that testosterone could induce human C-reactive protein gene expression in transgenic mice^15^. Testosterone is the major testicular hormone known for its anti-inflammatory effect^38^. For example, testosterone suppresses hepatic inflammation in an experimental acute cholangitis murine model^39^; testosterone also reduces TNFα and IL-1β pro-inflammatory cytokines^13,40^ and suppresses IL-6 production in murine bone marrow stromal cell lines^41^. Testosterone transduces its signal through binding to androgen receptors (AR). Interestingly, several studies have indicated that AR signaling might enhance inflammation under certain conditions. In macrophages, AR signaling enhances TNF-α expression^42^. It has also been reported that AR activation is associated with carcinogenesis in human hepatocellular carcinoma and pancreatic cancer with enhanced IL-6 signaling^43–45^. Thus, the AR signaling may enhance the IL-6-signaling strength in liver local inflammation, but not directly induced CRP expression in male mice.

One other possibility to explain the significantly high basal expression level in male hCRP-luc mice is the differences in hCRP promoter activation by transcription factors in murine and human cells. Differences between human and mouse CRP gene cis-regulatory elements have been attributed to the different degree of CRP gene activation during APR between these two species. For example, human CRP promotor have both STAT3 and C/EBPβ binding site that can respond to IL-6 and IL-1 signalling while mouse CRP promotor has STAT3 but not C/EBPβ site^46^. The expression of hCRP in murine cells is dependent on the interaction between trans-acting mouse transcription factors and cis-acting human regulatory sequences. The presence of different trans-activating factors in mouse, especially those have differential expression pattern from human, might contribute to the different basal expression of hCRP in mice. For example, the expression level of HNF-1α, a trans-acting mouse transcription factor on hCRP promoter, is 2-fold higher in mouse liver than in human liver^47^. However, the extent of how each and every one of these human regulatory elements is regulated in mouse cells is relatively unknown. In addition, hCRP promoter activation in mouse liver may also be regulated by sex-specific mouse liver gene expression^48^. The *CRP* was the only annotated human gene in the 181-kb BAC transgene fragment we introduced. Our CRP-Luciferase transgene was dramatically larger than the one used in previous human CRP transgenic mice^12,27^. Therefore, our transgene was likely to include most of the cis-regulatory transcription elements that could regulate CRP transcription in humans^32,49–52^. In this regard, our hCRP-Luc mice provide a valuable model to study how gender specific genes regulate hCRP gene expression.

In summary, we generated a highly sensitive mouse that reported hCRP gene expression instead of serum protein measurement *in vivo*. The induction of liver bioluminescence in the animal was IL-6-mediated, highly sensitive to multiple TLR ligands, and feasible for repeated endotoxin stimulations. The female hCRP-Luc mice is a potential non-invasive reporter tool for monitoring APR.

## Methods

### Animals

An IRES-Luc cassette was inserted into the human BAC clone RP11-158E22 (BAC/PAC resources center, Children’s Hospital Oakland Research Institute) with human CRP gene (Fig. 1a). The IRES-Luc cassette contained the luciferase cDNA (from pGL3-basic plasmid, Promega) and the IRES element (from pLVX-IRES-tdTomato, Clontech Laboratories). In addition, the IRES-Luc sequences were followed by bovine growth hormone polyadenylation (BGH-pA) signal (from pcDNA3.1 plasmid, Invitrogen). The cassette was constructed and introduced into the human BAC clone RP11-158E22 using a Red/ET recombination kit (Gene Bridges). The transgenic BAC construct was isolated and linearized by Not I, and a 181-kb transgene fragment was purified by phenol-chloroform extraction for subsequent pronuclear injection in mouse embryos. The CRP-Luciferase transgene was located in the centre, with 64 kb of 5’ flanking sequence and 112 kb of 3’ flanking sequence. C57BL/6-Tg(hCRP-IRES-Luc) mice (hCRP-Luc mice) were generated by the Genetic Engineered Murine Model Service (GEMMS) and deposited in the Rodent Model Resource Center (RMRC13210, RMRC; http://www.nlac.org.tw/RMRC/). hCRP-Luc mice were bred and held at the National Laboratory Animal Center (NLAC), National Applied Research Laboratories (NARL). Hemizygous hCRP-Luc mice were bred with wildtype C57BL/6JNarl mice purchased from NLAC. The genomic DNA from hCRP-Luc offspring were collected and screened by PCR. Luc4637F (Forward, TCACGCAGGCAGTTCTATGAGG) and Luc5147R (Reverse, GAGGTGGACATCACTTACGCTGAG) primers were used to amplify a 510-bp fragment of the luciferase transgene. Mice at 8–16 weeks of age were used for experiments.

### Approval

All animal procedures were reviewed and approved by the Institutional Animal Care and Use Committee (IACUC) of National Laboratory Animal Center (NLAC; protocol numbers NLAC-107-M-005). Post approval monitoring (PAM) programs were implemented during the research. All methods were performed in accordance with the relevant guidelines and regulations.

### *In vivo* induction of acute phase responses

Several TLR ligands and standard endotoxin were used in this study. Lipopolysaccharides (LPS) from Escherichia coli 0111:B4 were purchased from Sigma-Aldrich (L2630). LPS stock (2 mg/mL) was prepared with endotoxin-free saline (Taiwan Biotech). High-molecular weight polyinosinic-polycytidylic acid (poly(I:C)) was purchased from InvivoGen (tlrl-pic). Stock solution (1 mg/mL) of poly(I:C) in endotoxin-free saline was incubated for 10 min at 65–70 °C and cooled at room temperature for 1 h for proper annealing. Zymosan (Sigma-Aldrich Z4250), a polysaccharide connected by β-1,3-glycosidic linkages from Saccharomyces cerevisiae, was suspended in endotoxin-free saline (10 mg/mL). The zymosan suspension was boiled for 10 min at 100 °C and then kept on ice before use. All TLR ligand stock solutions were diluted to working concentrations (described in figure legends) with the same batch of endotoxin-free saline before injection.

To avoid endotoxin contamination during ligand preparation, poly(I:C) and zymosan were prepared using the same batch of endotoxin-free saline. The endotoxin concentration in poly(I:C) solution was <0.005 EU/mL as determined by Biolasco, Taiwan, using Endosafe Nexgen–PTS Test (PTS150, The Charles River Laboratory). The endotoxin level of zymosan could not be determined because zymosan itself could give false positive results.

E-Toxate Endotoxin standard was purchased from Sigma-Aldrich (E8029) and diluted serially with endotoxin-free saline. Recombinant murine IL-6 (1 μg/mouse, Catalog Number: 216-16, PeproTech) was injected intraperitoneally to induce bioluminescence of the female hCRP-Luc mice. For IL-6 neutralization test, anti-IL-6 monoclonal antibody (200 μg/mouse, Clone: MP5–20F3, Bio X cell) or isotype-matched irrelevant mAb (200 μg/mouse, Clone: HRPN, Bio X cell) was simultaneously co-injected with LPS *via* intraperitoneal route.

### Bioluminescence analysis

The bioluminescence analyses of each mouse were performed at indicated times after TLR ligand injection. The mouse was injected intraperitoneally with 200 μl D-luciferin (15 mg/mL, Cat. L-123-250, GoldBio). After 10 minutes, the mouse was then anesthetized with isoflurane (3% vaporized in O_2_) and the bioluminescence signals were captured and analyzed using the *In Vivo* Imaging System (IVIS, IVIS 100, Caliper) or AmiHT (Spectral Instruments Imaging). Total flux of bioluminescence signals was analyzed from a fixed region-of-interest (ROI) over the abdomen using Living Image software (Caliper, for IVIS-collected images) or Aura image software (Caliper, for AmiHT-collected images).

### Biomarker analysis

Peripheral blood samples were obtained from facial submandibular veins of mice using Golden Lancets (4 mm, Medipoint), and collected into a BD Microtainer Capillary Blood Collector (Cat. 365967). After centrifugation at 400 × *g* for 5 mins. Sera were harvested and frozen in a −20 °C freezer before analysis. Mouse APP concentration including serum amyloid protein/pentraxin-2 (SAP), adipsin, alpha-1 acid glycoprotein (AGP), and alpha-2 macroglobulin (A2M) were determined using a Multiplex kit (Millipore; MAP2MAG-76K-04) under standard procedures and analyzed by Luminex® 100 system. Mouse IL-6 and TNF were determined using the BD CBA flex set system (Cat. 558301 and 558299) and analyzed by FCAP Array software (BD Bioscience).

## Statistics

Analyses of the bioluminescence, acute phase protein, and cytokine productions were performed using one-way ANOVA with post-hoc Tukey HSD (Honestly Significant Difference) test. The LAL standard endotoxin-induced, dose-dependent bioluminescence in female hCRP-Luc mice was analyzed by linear regression method. All statistics were calculated using GraphPad Prism version 6 for Windows. The results were statistically significant when *p* < 0.05.

## Supplementary information

Dataset 1
