# Supplementary material for: A reporter mouse for non-invasive detection of toll-like receptor ligands induced acute phase responses
Source: Sci Rep. 2019 Dec 13;9:19065. doi: 10.1038/s41598-019-55281-w (PMC6910947; doi:10.1038/s41598-019-55281-w)
Supplement: Supplementary file 1 — Dataset 1 [file 41598_2019_55281_MOESM1_ESM.pdf]

SUPPLEMENTARY INFORMATION

Full title: A reporter mouse for non-invasive detection of toll-like receptor ligands induced acute phase responses

Authors: Chun-Fang Huang; Shang-Yi Chiu; Hung-Wen Huang; Bing-Ho Cheng; Hsiu-Min Pan; Wei-Lun Huang; Hsiao-Hui Chang; Chia-Chi Liao; Si-Tse Jiang; Yu-Chia Su*

Affiliations: National Laboratory Animal Center, National Applied Research Laboratories, Taipei, Taiwan

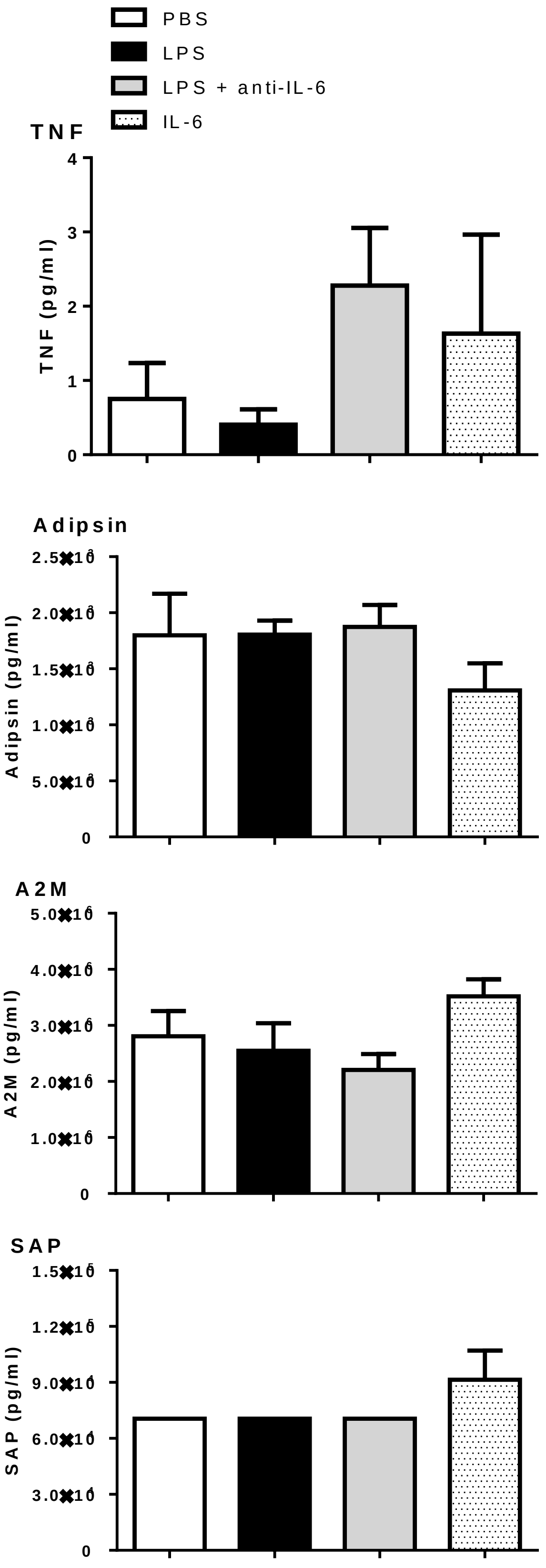

Figure-S1. LPS does not induce expressions of TNF and other acute phase protein. hCRP-Luc mice were intraperitoneally injected with sterile PBS, LPS (0.1 μg/mouse), IL-6 (1 μg/mouse), or LPS (0.1 μ g/mouse)/anti-IL-6 neutralized mAb (200 μg/mouse). Sera were collected for determination of adipsin, alpha-2 macroglobulin (A2M), serum amyloid P component (SAP) and TNF as described in Materials and Methods. Data are shown by Means ± SD (n=4-5/group).
